# A Case of Maternal Sepsis and Fetal Demise Associated with *Streptococcus pseudoporcinus*

**DOI:** 10.1155/2019/4309191

**Published:** 2019-12-04

**Authors:** Stephanie L. Pierce, Dena R. Shibib, Denise Robison, Rodney K. Edwards

**Affiliations:** ^1^Department of Obstetrics and Gynecology, College of Medicine, University of Oklahoma Health Sciences Center, Oklahoma City, OK, USA; ^2^Department of Pathology, College of Medicine, University of Oklahoma Health Sciences Center, Oklahoma City, OK, USA; ^3^Department of Pathology, Oklahoma City VA Health Care System, Oklahoma City, OK, USA; ^4^OU Medicine Microbiology Laboratory, Oklahoma City, OK, USA

## Abstract

*Streptococcus pseudoporcinus* has recently been described in relation to its colonization of the female genitourinary tract. Since prior reports have linked *S. pseudoporcinus* only with minor morbidities, the organism previously has not been considered to be a cause of serious puerperal infections. A 41-year-old gravida 2, para 1-0-0-1 presented with abdominal pain and intrauterine fetal demise. A beta hemolytic *Streptococcus* was isolated from her placenta, endometrium, urine, and two blood culture sets. The isolate was a *Streptococcus pseudoporcinus*, which colonizes the female genital tract and can resemble *Streptococcus agalactiae*. This case demonstrates that *S. pseudoporcinus* is a potential cause of severe maternal and fetal morbidity and mortality.

## 1. Introduction

Puerperal infection (e.g., chorioamnionitis, endometritis, or cesarean wound infection) is an important cause of maternal morbidity and mortality. Some of the most common organisms implicated in puerperal infection are *Escherichia coli*, other Enterobacteriaceae, *Staphylococcus aureus*, group A *Streptococcus*, and *Streptococcus agalactiae *(aka group B *Streptococcus*) [1]. Another member of the *Streptococcus* species, *Streptococcus pseudoporcinus*, recently has been found to colonize the female genitourinary tract [2, 3]. *S. pseudoporcinus *is noted to have biochemical characteristics similar to those of *S. agalactiae* and is therefore difficult to differentiate from *S. agalactiae* by traditional biochemical means [3]. As such, it is thought that the incidence of *S. pseudoporcinus* is likely underestimated due to misclassification as *S. agalactiae*. Prior case reports have linked *S. pseudoporcinus* with clinical infections including pyometra in a postmenopausal woman [4]. However, to our knowledge there have been no reports of major obstetric morbidity associated with this organism. Here, we discuss the case of a patient who presented with sepsis and fetal demise secondary to systemic infection with *S. pseudoporcinus*.

## 2. Case

A 41-year-old gravida 2, para 1-0-0-1 with no prenatal care presented to the Emergency Department (ED) with abdominal pain and was found to have a singleton fetal demise, ultrasound biometry consistent with 34 weeks gestational age. She had a past medical history significant for chronic hypertension. Her first pregnancy was uncomplicated and resulted in a term cesarean delivery of a liveborn infant. Upon presentation she was found to be in labor, with ruptured membranes and cervical dilation of 7 centimeters. Her exam was also notable for tachycardia and leakage of purulent fluid from the cervical os. She was afebrile, and labs were significant for a white blood cell count of 29,000 per mL and a serum lactate of 2.4 mmol/L. She received one dose each of intravenous piperacillin/tazobactam and cefazolin in the ED. Intravenous ampicillin and gentamicin were initiated in labor and delivery for a diagnosis of chorioamnionitis. She was also diagnosed with preeclampsia with severe features, and intravenous magnesium for seizure prophylaxis was administered.

Her labor progressed without augmentation, and she underwent spontaneous vaginal delivery of a stillborn infant. The patient declined autopsy and genetic testing for the infant.

After delivery, examination of the infant revealed no gross morphologic abnormalities. Placental pathology showed evidence of acute necrotizing chorioamnionitis and acute umbilical vasculitis, as well as mild decidual vasculopathy, consistent with maternal hypertension.

A beta hemolytic *Streptococcus* was isolated from the patient's urine, placenta, endometrium, and two blood culture sets. Lancefield grouping for *Streptococcus* groups A, B, C, F, and G were negative (Streptex, Remel, Lenexa, KS). Biochemical studies included a positive pyrrolidonyl aminopeptidase (PYR) test and a negative catalase reaction. The isolates were resistant to optochin. API (API 20 Strep, BioMerieux, Durham, NC) identification revealed S*treptococcus agalactiae* (biotype number 3063214) with 99.8% confidence at 24 hours. Identification of all isolates by matrix-associated laser desorption/ionization time-of-flight (MALDI-ToF; Bruker Daltronics, Billerica, MA) yielded *Streptococcus pseudoporcinus*, using the research use only (RUO) database (version 3.2.12.2) with log scores for each specimen ranging from 1.86 to 2.07. A blood culture isolate sent to a reference laboratory for bacterial 16S rRNA sequencing confirmed the identification of *Streptococcus pseudoporcinus*, with 100% identity of the first 467 base pairs of the 16S sequence to *Streptococcus pseudoporcinus* LQ 940–04. Isolates from the blood and placenta tested susceptible to ampicillin, clindamycin, erythromycin, penicillin, and vancomycin.

The patient recovered well in the postpartum period, remaining afebrile with normal vital signs and a normal serum lactate. On postpartum day 3, she was transitioned from intravenous ampicillin and gentamicin to oral amoxicillin. She left against medical advice on postpartum day 4. Written informed consent permitting the use of the patient's case for education and publication was obtained during her hospital stay.

## 3. Discussion

*S. pseudoporcinus* was first reported as a separate species in 2006, based on phenotypic analysis and gene sequencing of isolates that originated from the female genitourinary tract and were previously classified as *Streptococcus porcinus*. *S. porcinus* was first described in 1984 as mostly originating from swine [2]. Both species bear great resemblance to *S. agalactiae*, though *S. pseudoporcinus* typically demonstrates a wider zone of beta hemolysis. *S. porcinus* and *S. pseudoporcinus* may cross react with group B antisera, although some isolates do not [3]. Notably, in this case our isolates did not react with the Streptex group B reagents. In 2012, a review of 97 isolates previously identified as *S. porcinus* was performed using gene sequencing as well as conventional biochemical testing. It was found that 19 isolates were *S. porcinus*, while the remaining 78 isolates were actually *S. pseudoporcinus* [5]. Based on these and other similar findings, *S. pseudoporcinus* should be suspected in isolates that are phenotypically similar to *S. agalactiae* but demonstrate a wider zone of beta-hemolysis.

The similarity of *S. pseudoporcinus* to *S. agalactiae* raises concern that *S. pseudoporcinus* may be underreported. One study examining the prevalence of *S. pseudoporcinus* detected the organism in 1.6% of vaginorectal cultures screening for group B *Streptococcus* (GBS) [6]. It is therefore likely that some proportion of *S. pseudoporcinus* is misclassified as *S. agalactiae*, particularly in cultures of the female reproductive tract obtained specifically for the purpose of determining whether the patient is colonized with *S. agalactiae*.

Previous reports have demonstrated clinical infections associated with *S. pseudoporcinus *[4, 7, 8], but to our knowledge there have been no reports of this organism in association with severe obstetric morbidity. One recent report describes a patient with a vaginorectal culture at 39 weeks that grew a beta-hemolytic organism that was identified as *S. pseudoporcinus*, however there were no associated adverse maternal or neonatal outcomes in that pregnancy [9]. In addition, a study by Gaudreau et al. found that of 15 women colonized with *S. pseudoporcinus*, three patients developed chorioamnionitis, and two of those had preterm deliveries [10]. Furthermore, Grundy and colleagues recently demonstrated an increased risk of PPROM and preterm labor in a cohort of patients colonized with *S. pseudoporcinus *compared to those colonized with GBS [6]. However, one cannot conclude from these studies that the organism was the causative agent in those adverse outcomes. In contrast to the previous reports, our case demonstrates an instance of significant maternal morbidity (sepsis) and fetal demise in the setting of systemic maternal (and likely fetal) *S. pseudoporcinus* infection. Interestingly, Martin et al. previously reported a case of fetal demise associated with *S. porcinus* [11], however this was prior to the identification of *S. pseudoporcinus* as a separate organism in 2006. Therefore, it is possible the organism in that case may have been misidentified at the time as *S. porcinus*.

Currently there are no clinical guidelines that recommend screening or prophylaxis for *S. pseudoporcinus *in pregnancy. In a study of the incidence and epidemiology of *S. pseudoporcinus *[3], Stoner et al. conclude that there is a need to identify *S. pseudoporcinus* separately from *S. agalactiae* in order to avoid the unnecessary administration of antibiotics in pregnancy. However in light of our case which demonstrates significant morbidity related to *S. pseudoporcinus* infection in pregnancy, if this organism were misidentified as *S. agalactiae* and a patient received unnecessary intrapartum antibiotic prophylaxis, prophylaxis would pose minimal increased risk to the patient and could theoretically prevent some cases of puerperal infection caused by *S. pseudoporcinus. *If this organism is found to be the cause of morbidity in other cases, then this may have implications for screening and treatment guidelines in pregnancy.

The clinical necessity of specifically testing for *S. pseudoporcinus* in vaginorectal samples is questionable, since it remains unclear whether serious obstetric morbidity related to this organism occurs frequently enough to justify routine testing and because misclassification as *S. agalactiae* may occur. Although the exact clinical significance of *S. pseudoporcinus* remains to be seen, our case demonstrates that it is a potential cause of serious puerperal infection. If there are other reports of puerperal infections with this organism, its significance and prevalence in genital tract flora might warrant further investigation.
